# Barriers and strategies for recruiting participants who identify as racial minorities in musculoskeletal health research: a scoping review

**DOI:** 10.3389/fpubh.2023.1211520

**Published:** 2023-08-02

**Authors:** Denise Le, Rachel D. Almaw, Daniel Rinaldi, Natasha K. Ivanochko, Sheereen Harris, Ashley Benjamin, Monica R. Maly

**Affiliations:** ^1^Department of Biology, University of Waterloo, Waterloo, ON, Canada; ^2^School of Public Health Sciences, University of Waterloo, Waterloo, ON, Canada; ^3^Department of Kinesiology and Health Sciences, University of Waterloo, Waterloo, ON, Canada; ^4^Department of Kinesiology, McMaster University, Hamilton, ON, Canada; ^5^Department of Chemistry, University of Waterloo, Waterloo, ON, Canada

**Keywords:** race, ethnicity, diversity, minority, research strategies, recruitment, patient participation, clinical research

## Abstract

**Objective:**

Visible minorities are disproportionately affected by musculoskeletal disorders (MSD) and other diseases; yet are largely underrepresented in health research. The purpose of this scoping review was to identify barriers and strategies associated with increasing recruitment of visible minorities in MSD research.

**Methods:**

Electronic databases (MEDLINE, EMBASE, CINAHL, and PsycInfo) were searched. Search strategies used terms related to the concepts of ‘race/ethnicity’, ‘participation’, ‘research’ and ‘musculoskeletal’. All research designs were included. Two reviewers independently screened titles and abstracts, completed full-text reviews, and extracted data. Papers that did not focus on musculoskeletal research, include racial minorities, or focus on participation in research were excluded. Study characteristics (study location, design and methods; sample characteristics (size, age, sex and race); MSD of interest) as well as barriers and strategies to increasing participation of visible minorities in MSD research were extracted from each article and summarized in a table format.

**Results:**

Of the 4,282 articles identified, 28 met inclusion criteria and were included. The majority were conducted in the United States (27 articles). Of the included studies, the groups of visible minorities represented were Black (25 articles), Hispanic (14 articles), Asian (6 articles), Indigenous (3 articles), Middle Eastern (1 article), and Multiracial (1 article). The most commonly cited barriers to research participation were mistrust, logistical barriers (e.g., transportation, inaccessible study location, financial constraints), and lack of awareness or understanding of research. Strategies for increasing diversity were ensuring benefit of participants, recruiting through sites serving the community of interest, and addressing logistical barriers.

**Conclusion:**

Understanding the importance of diversity in MSD research, collaborating with communities of visible minorities, and addressing logistical barriers may be effective in reducing barriers to the participation of visible minorities in health research. This review presents strategies to aid researchers in increasing inclusion in MSD-related research.

## Introduction

Musculoskeletal disorders (MSDs) are one of the leading causes of disability in Canada and affect 11 million (27.8%) Canadians; a prevalence projected to increase to 15 million by 2031 (1, 2). One of the most common MSDs is arthritis (3). Arthritis refers to health conditions that produce inflammation of joint tissues. There are over 100 forms of arthritis, with the most common including osteoarthritis (OA), rheumatoid arthritis, spondyloarthropathies and lupus (4). Arthritis creates more pain, depression, immobility, disability and unemployment than any other chronic condition (4, 5).

Race is a key risk factor for greater prevalence and severity of arthritis and other MSDs. Race is a social construct that reflects social, cultural and environmental experiences that can have biological implications on health (6–8). Visible minorities, defined in Canada as “persons, other than Aboriginal peoples, who are non-Caucasian in race or non-white in color” (9) are disproportionately impacted by MSDs including arthritis. In Canada, individuals identifying as Indigenous experience the highest prevalence of arthritis compared to any other racial group (10). In the United States, Black and Indigenous patients with arthritis experience worse pain intensity than White counterparts; yet are less likely to undergo joint replacement surgery and more likely to be recommended non-specific treatments such as opioids (8, 11, 12). This evidence emphasizes that health disparities among visible minorities are multifactorial and are not fully understood (13). In the context of systemic racism, interpersonal racism, colonialism, and implicit bias (14, 15), race likely influences health outcomes through multiple mechanisms: culturally appropriate and safe services, access to care, quality of care, and need of care due to stressors from psychosocial or environmental factors (8, 14, 15).

Underrepresentation of visible minorities in research contributes to these race-based health disparities by creating critical gaps in understanding the true underlying mechanisms and impacts of arthritis and other MSDs (13, 16–18). First, there is a paucity of research on the influence of race on health outcomes (8), which creates a gap in understanding how factors associated with race can be addressed to improve the identification and treatment of MSDs. Second, current health research includes samples of either unknown race, or predominantly White race, limiting our understanding of the generalizability of the findings. As of 2016, visible minorities make up 22.3% of Canadians (19); yet samples in health research rarely reflect this proportion of visible minorities. A scoping review by Khan and colleagues found that only 5 out of the 99 Canadian health studies examined used nationally representative data to study health (18). Of key concern, poor generalizability of research findings means that the true efficacy of diagnostic, prognostic and treatment approaches in visible minorities remains largely unknown (18). It is crucial to directly address these gaps to thoroughly understand health determinants where disparities exist (6). For example, the Kellgren-Lawrence grade (KLG) system used to indicate OA disease severity was developed based on predominantly White samples. The KLG systematically scores OA severity lower than self-reported OA pain intensity in Black compared to White patients (11). In the context of this finding, it is not surprising that Black patients are less likely than White patients to receive proper pain management for OA (20). A study utilizing machine learning showed that using a racially diverse dataset to assess OA severity was more accurate in predicting pain intensity in patients whose race is not White compared to KLG or other algorithms using non-diverse samples (11). This finding shows that diversifying datasets increases the generalizability of findings; and the underrepresentation of visible minorities in MSD research has produced a major gap. Thus, ensuring the participation of visible minorities in health research has potential to address race-based health disparities in MSDs.

By summarizing the existing research exploring racial representation in musculoskeletal health research, this paper will provide researchers and clinicians with information necessary to better engage visible minorities for their participation in research. The purpose of this scoping review is to highlight the unique barriers to research participation experienced by visible minorities; and identify strategies to increase recruitment and retention of visible minorities in MSD research. The findings aim to provide readers with tangible strategies to improve racial diversity in MSD research studies.

## Materials and methods

A scoping review methodology was selected to examine the extent, range and nature of strategies used to engage visible minorities in MSD research from a heterogeneous set of studies. This report uses the methodological framework proposed by Arksey and O’Malley (21, 22) and modified by Levac (23). This scoping review did not critically appraise articles because articles included various research designs; e.g., narrative review to randomized clinical trial. This scoping review was not registered. A team of researchers were involved in identifying, extracting, and synthesizing data on barriers and strategies to engage visible minorities in musculoskeletal research, as recommended by the Arksey and O’Malley Framework (24).

### Search strategy

The following electronic databases were searched from March 9 to March 12, 2021: MEDLINE (Ovid) 1946-2021, EMBASE (Ovid) 1974-2021, CINAHL (EBSCOhost) 1976-2021, and PsycInfo (ProQuest) 1800-2021. We also manually reviewed reference lists of eligible articles. The search strategies used keywords and MeSH terms related to ‘race/ethnicity’, ‘participation’, ‘research’ and ‘musculoskeletal’ and the search was limited to the English language. Search strategies were peer-reviewed by an information specialist (J. Stapleton) prior to the final implemented search (Supplementary material S1).

### Eligibility

We included studies that were published from 1964 to March 12, 2021, focused on MSD conditions and addressed barriers or strategies for recruitment of visible minorities. All research designs were included. The year 1964 was selected to reflect the implementation of the Helsinki declaration. We excluded studies that were not related to visible minorities, did not address recruitment in research, did not involve participants with musculoskeletal disorders, animal studies, gray literature (information produced outside traditional commercial or academic publishing and distribution), and studies where the full-text could not be accessed.

### Selection process

**Figure 1:**
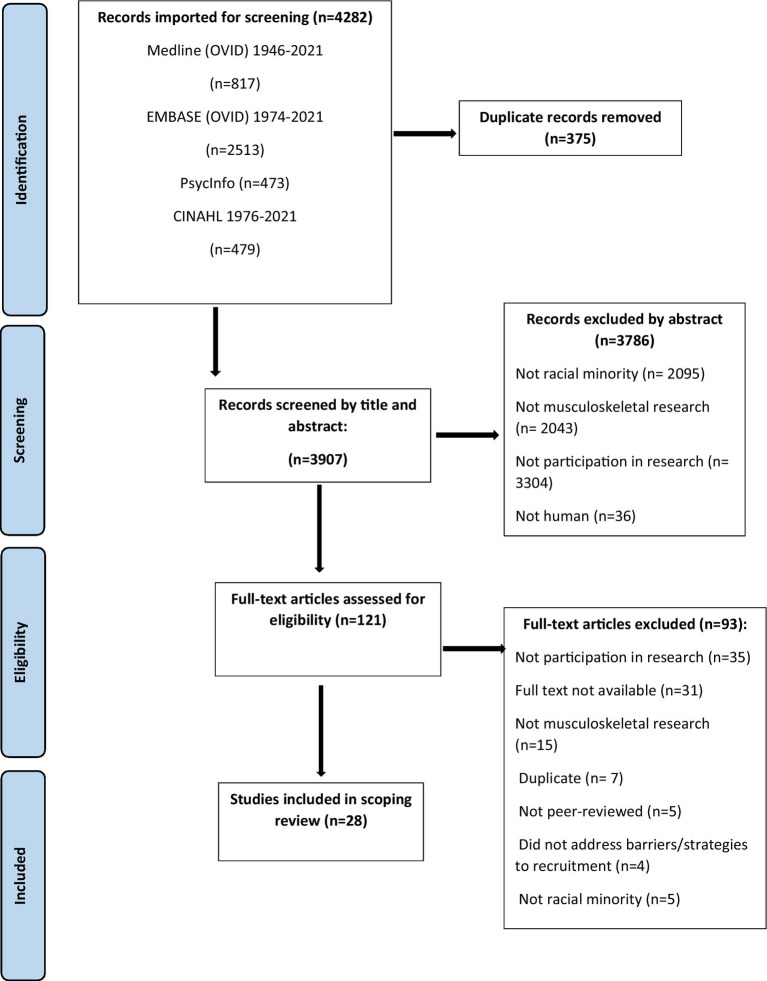
PRISMA flow chart.

An overview of the selection process is represented in Figure 1. Title and abstracts were independently reviewed by two authors (DL and RA; DL and DR) for inclusion. Between-rater agreement was high for DL and RA [absolute agreement = 88.1% and relative agreement (Cohen’s Kappa value) = 0.41]; and DL and DR [absolute agreement = 94.3% and relative agreement (Cohen’s Kappa value) = 0.45]. Where there were disagreements, authors met with a third team member (MM) to reach consensus. Next, full-texts were imported into a data management software package (Covidence, Melbourne, Australia) and independently reviewed by DL and RA, then verified by DR. Duplicates were automatically removed by Covidence and manually identified by DL and RA.

### Data extraction

The study team developed, pilot tested and revised a standardized form to chart data extracted from each study included in the scoping review. The following data were extracted: study location, design, and methods; sample characteristics (size, age, sex, and race); MSD of interest; and barriers and strategies to participation. These data were independently extracted and charted by two separate reviewers, where any conflicts were then resolved and verified by a third reviewer.

### Synthesis of results

First, data regarding the characteristics of each study, sample characteristics, and disorder(s) of interest were collated and presented in a table to represent the scope and breadth of the evidence. Second, barriers to the participation of visible minorities in research identified in each study were compiled, then organized into major themes that emerged. Topics within these themes were presented in the order of frequency that was identified among the included studies. Strategies implemented in these studies to directly address these barriers were presented, again in the order of frequency. Led by DL and RA, all authors participated in establishing these major themes that emerged from the included studies. These themes on barriers and the corresponding strategies were summarized in a table.

## Results

### Study characteristics

Of the 4,282 articles retrieved, 28 were included (Table 1). Almost all were located in the United States (US) (27 articles) with one in Australia. MSDs studied were systemic lupus erythematosus (SLE)^1^ (7 articles), osteoporosis (5 articles), arthritis (4 articles), rheumatoid arthritis (RA) or other rheumatic diseases (4 articles), lupus (3 articles), chronic back pain (2 articles), OA (2 articles), multiple or general MSD pain/illness (2 articles) and gout (1 article). Study designs included qualitative (9 articles), secondary analysis (8 articles), cross-sectional (4 articles), systematic or literature review (4 articles), randomized controlled trial (2 articles), and exploratory (1 article). A variety of methodologies were used, including analysis of recruitment methods/yield (6 articles), interviews (6 articles), questionnaires (5 articles), focus groups (5 articles), and surveys (5 articles). Races included Black (25 articles), Hispanic (14 articles), Asian (6 articles), Indigenous (3 articles), Middle Eastern (1 article), and Multiracial (1 article).

**Table 1:** Summary of study characteristics and methods.

Author	Country/location	Sample characteristics	Race	Disease under study	Study design	Methods
Anjorin and Lipsky (25)	N/A	N/A	Black	SLE	Systematic review	Systematic literature review
Arriens et al. (26)	Oklahoma, US	*n* = 23 (*F*: 20 M: 3); White (34.8%, *n* = 8), Black (30.4%, *n* = 7), Hispanic/Latino (21.7%, *n* = 5), Indigenous (13%, *n* = 3); Age: 21-72	Black, Hispanic, Indigenous, White	SLE	Qualitative	Focus Groups
Blank et al. (27)	Minneapolis, Birmingham, Pittsburgh, Palo Alto, San Diego	*n* = 5,995, Black (4%, *n* = 244), Asian (3%, *n* = 191), White(89.4%, *n* = 5,362); Age: ≥ 65	Black, Asian, White	Osteoporotic Fractures	Secondary analysis of observational study	Questionnaire
Brady et al. (28)	Sydney, Australia	*n* = 48, Mandaean (33.3%, *n* = 16), Assyrian (33.3%, *n* = 16), Vietnamese (33.3%, *n* = 16); Age: ≥18 years	Middle Eastern (Mandaean*, Assyrian), Southeast Asian (Vietnamese)	Chronic MSK Pain	RCT	Questionnaire
Callahan et al. (29)	North Carolina, US	*n* = 285, Hispanic (100%, *n* = 285); Age: 21	Hispanic	Arthritis	Qualitative	Survey, Questionnaire, Focus Groups, Interviews
Der Ananian et al. (30)	US	*n* = 205, White (58.1%, *n* = 119), Black (41.9%, *n* = 86); Age: 50	Black, White	Arthritis	Cross-Sectional study	Survey
Drenkard et al. (31)	Southeastern US	*n* = 168 (*F*: 168), Black (100%, *n* = 168)	Black	SLE	Qualitative	Survey
Dunbar-Jacob et al. (32)	US	initial screening: *n* = 961, Black (11%, *n* = 101), White (89%, *n* = 860); final screening: *n* = 188, Black (6%, *n* = 12), White (94%, *n* = 176); Age: ≥ 30	Black, White	RA	Qualitative	Phone Interview
Figaro et al. (33)	US	*n* = 94 (*F*: 84, M: 10), Black (100%, *n* = 94); Age: ≥ 50	Black	OA	Qualitative	Interviews
Gaskin et al. (34)	US	*n* = 21 (*F*: 21), Latina (57.2%, *n* = 12), Black (42.8%, *n* = 9); Age: 45	Latina, Black	Arthritis	Qualitative	Focus Groups
Groupp et al. (35)	Portland, Oregon, US	*n* = 120, White (84%, *n* = 100), Black (16%, *n* = 20); Age: 60	Black, White	Chronic Low Back Pain	RCT	Questionnaire, Survey
Lee et al. (36)	San Diego, California, US	*n* = 191, Hispanic (57%, *n* = 109), White (25%, *n* = 48), Asian (12%, *n* = 23), Black (6%, *n* = 11)	Hispanic†, Asian, Black, White	RA	Cross-sectional study	Survey
Lee et al. (37)	US	*n* = 23, White (48%, *n* = 11), Hispanic (30%, *n* = 7), Black (13%, *n* = 3), Indigenous (4.5%, *n* = 1), Asian (4.5%, *n* = 1); Age: 34-84	Hispanic, Black, Indigenous, Asian, White	RA, OA, Multiple Illnesses	Qualitative	Focus Groups
Lim et al. (38)	Atlanta, Georgia, US	*n* = 18 (*F*:18), White (66.6%, *n* = 12), Black (33.3%, *n* = 6); Age: ≥ 27	Black, White	SLE, Lupus Nephritis	Secondary analysis of clinical trial simulations	Interview, Questionnaire
Lima et al. (39)	N/A	*n* = 1892 from 7 articles (5 focused on Black, 1 focused on Hispanic participants)	Black, Hispanic	Lupus, RA	Systematic review	N/A
Marquez et al. (40)	Olmsted County, Minnesota, US	*n* = 1,487 (*F*: 848, *M*: 639); 47%, *n* = 699 non-white; Southeast Asian Immigrants (26.6%, *n* = 396: 172 Vietnamese, 171 Cambodian, 53 Laotian), Hispanic (6%, *n* = 88), White (53% *n* = 788); Mean age: 55.1	Southeast Asian (Vietnamese, Cambodian, Lao), Black (Somali), Hispanic, White	Osteoporosis	Secondary analysis of observational study	Analysis of recruitment methods and expenses
Miller et al. (41)	Baltimore, Maryland, US	*n* = 77, Black (48%, *n* = 37), White (32%, *n* = 24), Indigenous/Asian/Pacific Islander (9%, *n* = 7), Unknown race (12%, *n* = 9); Age: ≥18	Black, White, Indigenous/Asian/Pacific Islander, Unknown	Gout, Hypertension	Secondary analysis of a RCT	Analysis of recruitment yield and cost effectiveness across different recruitment methods
De Brey et al. (42)	US	Urban, monolingual, Spanish-speaking participants; study 1: *n* = 161; study 2: *n* = 245	Hispanic	Arthritis	Secondary analysis	Systematic comparison of study recruitment methods
Rodgers et al. (43)	Charleston, US	*n* = 19; 9 patients (2 White F, 1 Hispanic F, 5 Black F, 1 Black M), 4 rheumatologists, 4 community members, 5 tour guides	Black, Hispanic, White	SLE	Qualitative Ethnographic Study	Interviews
Sheikh et al. (44)	US	N/A	Black, Hispanic	SLE	Literature review	N/A
Townley et al. (45)	New York City, US	*n* = 90, White (33.3%, *n* = 30), Hispanic (33.3%, *n* = 30), Black (33.3%, *n* = 30); Mean age: 60	Black, Hispanic, White	Chronic back pain	Cross-sectional survey	Interviews
Unson et al. (46)	Connecticut, US	*n* = 16, Black (*n* = 16); Mean age: 75	Black	Osteoporosis	Exploratory study	Focus groups
Unson et al. (47)	Connecticut, US	*n* = 904, White (59.5%, *n* = 538), Black (26.8%, *n* = 242), Hispanic (13.7%, *n* = 124)	Black, Hispanic, White	Osteoporosis	Secondary analysis of a clinical trial	Analysis of productivity of recruitment methods
Unson et al. (48)	Connecticut, US	*n* = 904, Black (26.8%, *n* = 242), Hispanic (13.7%, *n* = 124), White (59.5%, *n* = 538); Mean age: 75	Black, Hispanic, White	Osteoporosis	Secondary analysis of a clinical trial	Statistical analysis of demographic, enrollment, and eligibility data
Vina et al. (49)	US	*n* = 343, Black (47.5%, *n* = 163), White (52.5%, *n* = 180), Age: ≥ 18	Black	SLE	Cross-sectional study	Phone interviews and medical record reviews
Wallen et al. (50)	US	*n* = 15 (patients and community leaders), Black (47%, *n* = 7), Hispanic (33%, *n* = 5), Asian/Pacific Islander (7%, *n* = 1), White (13%, *n* = 2), Multiracial (7%, *n* = 1); Mean age: 51	Black, Hispanic, Asian/pacific islander, multiracial, white	Rheumatic disease	Qualitative	Focus groups
Warren-Findlow et al. (51)	Illinois, US	*n* = 70, Black (66%, *n* = 46), White (74%, *n* = 52)	Black, white	Multiple chronic illnesses including arthritis	Secondary analysis	Analysis of factors related to recruitment and retention in a longitudinal intervention study
Williams et al. (52)	US	People with lupus, researchers, physicians, patient advocacy groups, government representatives; Non-white (57.5%)	Non-white (mostly Black)	Lupus	Review	Summary of conference

Sample sizes (*n*) represents participants diagnosed with the disease under study unless otherwise indicated. *Mandaean is an ethnoreligious group originating from the Middle East, and therefore would not be characterized as a distinct ethnicity or race. RCT, Randomized Controlled Trial; US, United States; F, Female; M, Male; SLE, Systemic Lupus Erythematosus; RA, Rheumatoid Arthritis; OA, Osteoarthritis; MSK, Musculoskeletal. †Analysis focused on specific race.

### Barriers and strategies to participation in research

Barriers and strategies identified in the included articles were organized into the following four themes: Participant Willingness; Participant Opportunity; Research Design; and Healthcare Provider. These themes are defined and described below. Table 2 summarizes the barriers and strategies identified among the 28 included articles.

**Table 2:** Barriers and strategies to visible minority participation in musculoskeletal research.

Theme	Barriers	Strategies
Participant willingness	Mistrust and fear of exploitation due to historical mistreatment of minorities in research (12 articles) (25, 32, 35–37, 39, 44, 46, 49–52) Lack of awareness or understanding about research (*n* = 11) (25, 26, 28, 35–38, 41, 44, 50, 52) Low health literacy, lack of access to scientific information, misconceptions and/or lack of understanding of what research entails Lack of personal benefit (7 articles) (26, 35, 36, 44, 45, 49, 51) Skepticism of treatment efficacy, possibility of receiving placebo, low expectations of benefits Program/treatment may not resonate with participants, disinterest in study Requirement to stop other treatments Disappointment from research outcomes (ineligibility, withdrawal, sparse feedback, do not capture lived experience) Language barriers (5 articles) (28, 37, 44, 45, 52) Mistrust amplified when English was not first language of participant Difficulty reading recruitment materials Fear of unknown side effects and risks (5 articles) (26, 35, 36, 39, 46) Stigma around disease or research from support system (4 articles) (25, 38, 43, 46) Informed consent and confidentiality (4 articles) (26, 37, 38, 52) Intimidated by informed consent, burdensome forms Confidentiality concerns Invasive procedures and screening processes (3 articles) (36, 46, 51) e.g., screening procedures, fear or inconvenience of giving biological materials like blood and urine (related to mistrust) Lack of support (from family and community) (2 articles) (38, 44) Lack of community support and exclusion of family members/support system in enrolment	Ensuring benefit to participants (9 articles) (26, 29, 35–37, 43, 45, 46, 52) Emphasizing potential for improved health, emphasizing altruistic benefits during recruitment and throughout study Compensation (transport, childcare costs, monetary payments) Access to thorough and free care like physician visits, tests, complementary screening for musculoskeletal conditions Tailoring interventions and/or programs to accommodate different functional abilities and cultures Offering opportunities for social support with peers with similar conditions Overcoming language barriers (6 articles) (29, 37, 40, 45, 47, 48) Bilingual research staff, employing bilingual advisors and using translated recruitment materials and research instruments Developing networks with community leaders in visible minority communities (5 articles) (e.g., clergy, senior centers, service organizations) (35, 40, 42, 50, 52) Forming relationships in communities with visible minorities through visits to sites, community talks, and easy phone access Encouraging visibility and involvement in community activities beyond research to optimize trust Reducing mistrust through communication (4 articles) (26, 31, 46, 50) Frequent communication between contact and participants (including follow up feedback) Building trusting relationships between participants and their communities, and culturally competent staff Be cognizant of discomfort during study Participate in community events to make research institutions accessible and familiar to minorities Engaging meaningfully throughout all or important stages of research design (4 articles) (34, 39, 50, 52) Receiving input from intended audience Partnerships with local patient advocacy groups Engaging community partners at all stages of research design Educating participants (4 articles) (37, 41, 46, 52) Participant education about research opportunities and expectations (especially those who do not speak English natively) Community and faith-based participatory research approaches, providing practical information and educational sessions Engaging patient support systems (3 articles) (28, 39, 50) Encouraging patient to consult community representatives and family members before consenting Option to bring a person of their choice to data collection Training research staff (3 articles) (29, 31, 52) Address and avoid implicit biases in participant engagement, outreach and recruitment of minorities Applying cultural and linguistic competence to all steps of the research process, creating culturally tailored programs Using clear and culturally relevant recruitment materials (3 articles) (31, 42, 48) Concise, positive tone, clear lay language, potentially bilingual Creating appropriate consent forms (2 articles) (39, 40) Straightforward and transparent consent forms provided to subjects in English and/or native language along with verbal explanations Providing flexibility in data collection (2 articles) (37, 52) Personalization in frequency and method of data collection Improving racial concordance among participants and researchers / having a diverse research team (2 articles) (25, 39) Identifying and recruiting patient ambassadors to serve as gatekeepers (1 article) (39)
Participant opportunity	Logistical barriers (e.g., location, transportation, time, childcare, financial concerns) (10 articles) (25, 26, 31, 32, 36, 38, 39, 44, 45, 51) Competing demands (e.g., childcare, work) Location of trial site (e.g., lack of access to site, lack of trials within proximity of provider, safety of location) Lack of referrals from primary care physician (6 articles) (25, 37, 39, 42, 44, 52) Limited access to healthcare or specialists (3 articles) (37, 42, 44)	Recruiting in communities/locations that predominantly serve races of interest (11 articles) (eg. Senior housing, community clinics) † (27, 29, 30, 33–35, 42, 45, 47, 48, 51) Direct mailing or use of mailing lists Active recruitment, community outreach, community talks Word of mouth or specifically inviting participants to refer a friend/family member that meets criteria Recruiting through community-based physicians or physicians that predominately serve visible minorities Contacting participants from related studies, prior studies, pre-existing social networks Advertising through newspapers with predominantly minority audiences Improving logistics for access to research (7 articles) (26, 31, 40, 45–47, 51) Ensuring flexibility in scheduling and locations and provision of accommodations/reimbursements to reduce logistical barriers Increasing awareness to potential participants (4 articles) (37, 41, 46, 52) Educating potential participants about research opportunities and expectations (especially those who do not speak English natively) Community and faith-based participatory research approaches, providing practical information and educational sessions Creating positive relationships between patients and providers (4 articles) (25, 39, 49, 50) Shared decision-making, positivity, and friendliness in patient-provider relationship. Building trust in patient-physician relationships and acknowledge unconscious biases/structural racism Training healthcare providers to provide referrals (3 articles) (25, 44, 52) Education or training interventions that are provider focused, partnership-based and patient-meditated Providing flexibility in data collection (2 articles) (37, 52) Offering personalization of data collection (in frequency, method), ensure data collection is convenient Collecting data on participant race and/or ethnicity
Research design	Eligibility criteria (e.g., due to comorbidities) disproportionately exclude visible minorities (4 articles) (32, 35, 47, 52) Lack of connections to community (2 articles) (26, 52) Lack of communication of research findings to participants or treating physicians Implicit biases of researchers (2 articles) (44, 52)	Developing personal contact with community leaders within visible minority communities (e.g., clergy, senior centers, service organizations) (5 articles) (35, 40, 42, 50, 52) Forming relationships in communities with visible minorities through visits, community talks, and phone access Assistance from community gatekeepers in implementation and rollout of recruitment to ensure concordance Encouraging visibility and involvement in community activities beyond research to optimize trust Eligibility criteria (4 articles) (28, 32, 39, 51) Thoughtful design of eligibility criteria to recognize ethnocultural complexities to prevent exclusion of visible minorities Meaningful engagement throughout all or important stages of research design (4 articles) (34, 39, 50, 52) Partnerships with local patient advocacy groups Engaging community partners at all stages of research design for input Reducing mistrust through communication (4 articles) (26, 31, 46, 50) Frequent two-way communication with participants and provider and incorporation of post-study feedback Building relationships between participants and their communities, to encourage familiarity and accessibility to visible minorities Training research staff to engage with participants without introducing biases during study and in recruitment (3 articles) (29, 31, 52) Applying cultural and linguistic competence to research and in all steps of the research process, creating culturally tailored programs Identifying and recruiting patient ambassadors to serve as gatekeepers (1 article) (39)
Healthcare provider	Lack of referrals/support from primary care physician (6 articles) (25, 37, 39, 42, 44, 52) Implicit bias of referring physicians and fear of potentially losing patients Lack of understanding of the research process (e.g., referral letters may be associated with coercion)	Creating positive relationships between patients and providers (4 articles) (25, 39, 49, 50) Shared decision-making, positivity, and friendliness in patient-provider relationship. Building trust in patient-physician relationships and acknowledge unconscious biases/structural racism Training research staff (3 articles) (25, 44, 52) Understanding and addressing personal, institutional and implicit biases through training (i.e., site initiation training) Education or training interventions that are provider focused, partnership-based and patient-mediated

#### Participant willingness

Participant willingness referred to a person’s inclination toward consenting to, or being ready, to engage as a participant in a research activity. Barriers included the following: mistrust, lack of awareness, lack of personal benefit, language, fear of unknown risks, stigma, confidentiality, invasive procedures, and lack of support. Mistrust was cited most frequently. In 2019, this mistrust was highlighted at a conference focused on recruiting underrepresented groups into clinical studies. The conference involved people with lupus, patient advocacy groups, physicians, trialists and social scientists. This conference identified a key issue contributing to lack of trust in research was historical abuses of non-white subjects by medical investigators (52). Knowledge and tangible benefits directly to participants supported willingness to participate in research among visible minorities. A qualitative study of serial focus groups with 16 Black women showed that those who articulated knowledge about their health condition and its treatment, and those who perceived personal benefit with fewer concerns of “being used,” were more likely to enroll in a clinical study (46). Across included studies, strategies identified to overcome these barriers included ensuring benefits to participants, addressing language barriers, building networks with community leaders, reducing mistrust, engaging visible minorities in research design, educating, implementing patient support systems, training staff on inclusion practices, tailoring recruitment materials and consent forms, providing flexible data collection, diversifying the research team, and engaging patient ambassadors. Providing participants with tangible benefits was the most common topic, with benefits to personal health, altruism, financial support, and social support as key examples. In a study conducted at the University of California, willingness to participate in a clinical trial among patients with rheumatoid arthritis (*n* = 144, 82 Hispanic and 36 Caucasian) were predicted by the following benefits: free blood test [odds ratio (OR) 3.66 (1.82-7.53)], more doctor visits [OR 3.39 (1.68-6.99)], earlier access to therapy [OR 2.95 (1.48-6.01)] and free treatment [OR 2.67 (1.33-5.44)] (36). Strategies to enhance understanding of the research process included providing free educational sessions with practical information and disease screening and community and/or faith-based participatory research.

#### Participant opportunity

Participant opportunity described the structure and circumstances of research that foster, or diminish, the ability of visible minorities to participate. Barriers included logistics, lack of referral, and limited access to healthcare or a specialist. Logistical concerns were the most frequently cited barrier. Challenges for potential participants included work and childcare demands, inaccessible study location and finances. For example, a simulated clinical trial (methods included informed consent; mock screening, drug dosing; debriefing interview) emphasized that transportation, financial issues, childcare and the need for strong community and online support were needed to facilitate participation of Black people in a clinical trial of drug therapy for lupus (38). Strategies to enhance participant opportunity included recruiting in communities of visible minorities, addressing logistical barriers, and improving awareness, positive healthcare provider-patient relationships, referrals, and flexibility. Callahan and colleagues integrated multiple of these strategies: 288 participants enrolled in a trial of a 6-week, evidence-based walking program for Hispanic adults with arthritis, where bilingual, trained research staff conducted site-specific recruitment practices within Hispanic spaces (e.g., churches, consulate) and aided in reading and completing study materials. At 6 weeks, 233 (82%) participants completed the follow-up measures (29).

#### Research design

Research design refers to decisions made in the focus, methodology and interpretation of research activities, spanning from defining study objectives to disseminating findings. Barriers identified in the included articles were eligibility criteria, lack of community connections and implicit biases. The most frequently cited barrier incorporated in research design was eligibility criteria that disproportionately excludes visible minorities from becoming a research participant. In a study of people with rheumatoid arthritis, Dunbar-Jacob and colleagues reported that Black potential participants were more likely to be excluded due to comorbid conditions compared White potential participants (22.4% versus 13.1%) (32). Strategies identified included connection with community leaders, thoughtful eligibility criteria, engagement of visible minorities throughout research design, communication, training researchers and patient ambassadors. Multiple approaches to foster connection with community leaders were identified, ranging from educational presentations, community activities outside of research, and formal partnership. For example, a community-based participatory research approach was used to explore how to conduct health behavior research for people with arthritis in an urban setting (50). Informal and formal discussions with researchers, community leaders and patients enabled sharing in expertise, decision-making and ownership of the research process. This approach emphasized that researchers must be open to change based on the input of community leaders and patients (50).

#### Healthcare provider

This theme referred to the knowledge, experience, biases, and actions of healthcare providers that can foster, or discourage, the participation of visible minorities in clinical research. Topics in this healthcare provider theme influence participant willingness and participant opportunity. The key barrier identified in this review was that visible minorities were less likely to receive referrals or support from their primary physician to engage in research. In a narrative review focused on lupus, Sheikh and colleagues highlighted literature citing that healthcare providers lack familiarity and access to clinical trials (e.g., study information, eligibility criteria, and principal investigators), may have implicit biases (e.g., beliefs about adherence to trial protocols, negative impact on relationships with patients), and lack the resources to counsel patients about clinical trials (e.g., time, connection with principal investigator, proximity to trial site) (44). Training healthcare providers was identified as a strategy. Not only can training overcome the barrier of familiarity and access to research opportunities, training may also create an inclusive environment and promote positive patient-provider relationships; for example, through shared decision-making and acknowledging then addressing biases.

## Discussion

This scoping review identified barriers that contribute to underrepresentation of visible minorities in MSD research, including mistrust, a lack of awareness of research activities, as well as inaccessible research practices. Strategies to overcome these barriers emphasized directly addressing participant willingness and opportunity; meaningful community engagement; research activities that bolster recruitment and address logistical barriers; and positive relationships of healthcare providers with visible minorities. This scoping review also highlighted that the vast majority of research regarding underrepresentation took place in the United States. While invaluable, the heterogeneity within and between minority groups and the uniqueness of healthcare and research environments within and between countries suggests the experiences summarized in this review may not be widely generalizable.

The most frequently cited barriers were mistrust, logistics, and lack of awareness or understanding of research. Mistrust among visible minorities from various racial backgrounds reflected multiple contributors, including a history of exploitation in clinical or research environments based on race, as well as negative personal experiences and opinions from trusted community members such as family. Mistrust was emphasized in Black participants, stemming from a history of exploitation in research such as the Tuskegee study (which involved purposefully and deceptively withholding antibiotics for syphilis among Black participants to document the natural history of this disease) (53). Mistrust was further reinforced by negative experiences in health care and prevailing discrimination and exploitation (53). Black participants were more likely to believe that research findings will reinforce negative stereotypes about their race and expose them to unnecessary risks (53). We anticipate that mistrust would also be a significant barrier for other groups that have experienced a history of exploitation and/or colonization, such as Indigenous Peoples in Canada. Goodman (54) highlighted that Indigenous Peoples in Canada who participated in research experienced mistrust of researchers, lack of transparency, lack of benefit to the community, and were subjected to questionable research practices (54). Several barriers such as stigma, fear of unknown risks, invasive procedures, and confidentiality concerns may also be associated with mistrust. Additionally, language barriers, lack of understanding, and barriers related to opportunity were commonly identified by Hispanic participants. Studies that aimed to recruit Hispanic participants prioritized language accessibility and employing bilingual research staff (29, 42).

Existing theories that underscore the barriers confronted by visible minorities in societal systems, including healthcare and academic institutions, can help researchers understand the need to implement strategies for improving diversity in MSD research. Critical Race Theory (CRT) and the Public Health Critical Race praxis (PHCRP) introduced by Ford (55) are useful to consider because these theories provide a set of principles to understand inequities and identify strategies to address or eliminate factors contributing to inequities in health outcomes (55). To truly address underrepresentation, researchers must make advances toward breaking down structural racism and racial inequities. To make these advances, researchers can consider (i) gaining understanding of the impact of implicit biases and the importance of diversity in clinical research; (ii) acting through adequate training of research staff and thoughtful research design (e.g., ensuring benefit to participants, recruiting through sites serving the community of interest, addressing logistical barriers through flexibility in scheduling and locations, and reimbursements); (iii) collaborating with communities to meet needs and translate findings and; (iv) developing meaningful community partnerships to reduce health disparities. For example, working with communities and involvement of community leaders in the research process was emphasized as an important strategy (35, 40, 42, 50, 52). These goals of understanding, acting, collaborating and meaningful partnership can be conceived as existing on a continuum, with engagement that progressively requires more involvement from underrepresented communities. Designing research specifically to reduce inequity requires meaningful partnerships with visible minorities, such as that achieved through participatory action research. It is not feasible to implement all of these strategies at once. Researchers can begin by collecting data on race within existing research and begin understanding implicit racial biases and the role of these biases on limiting research impact.

As researchers progress on the continuum of improving diversity in MSD research, partnering with visible minority communities may have the largest impact on recruitment and meaningful engagement of participants. Community-engaged research approaches include “a process of working collaboratively with groups of people who are affiliated by geographic proximity, special interests, or similar situations with respect to issues affecting their well-being” (56). This involves engagement through partnerships with community members throughout the research process (57), such as recruiting through community sites. Engagement can also reduce mistrust, ensure benefit to participants and their communities, improve awareness, and overcome logistical barriers. In a study observing over-researched Indigenous Peoples in Canada, participants reported feeling left uninformed of research outcomes and objectives after information was taken, and that benefits were preserved for audiences outside of the community being researched (54). Lack of personal or tangible benefits reflects inadequate communication between researchers and communities of visible minorities. Further, disinterest of potential participants in a study may reflect a lack of culturally-important input that would be valued by the community.

A key resource to operationalize strategies to improve representation of visible minorities in MSD research is the PROGRESS-Plus framework. PROGRESS-Plus (Place of residence, Race/ ethnicity/ culture /language, Occupation, Gender, Religion, Education, Socio-economic status, Social capital and “Plus” that includes other context-specific factors) identifies social determinants and how they may influence recruitment, experiences, and unique needs of visible minorities in research participation (58). Nonetheless, because little data were found, it is unclear whether these strategies could be effective among other visible minority groups or in other contexts (To further support researchers interested in improving representation, we list resources in Supplementary material S2). Future work to aid researchers and clinicians could include exploring the effectiveness of implementing strategies presented in this review, as well as in frameworks such as PROGRESS-Plus in directly addressing underrepresentation in MSD research.

### Limitations and future work

The included studies did not include a broad range of visible minority groups. Visible minorities represented were mainly Black and Hispanic. Due to underrepresentation of visible minorities in Canadian studies, we do not have sufficient data to fully reflect the experiences of visible minorities that live in Canada, including Indigenous, South Asian, and East Asian, among others. Although 3 studies included Indigenous participants, data derived from these participants were not presented separately from the remaining sample and therefore we were unable to identify perspectives specific to this group. Therefore, the results may not recognize barriers experienced by Indigenous Peoples that reflect interactions with healthcare. The systems of care for Indigenous Peoples of Canada, which historically involved segregated healthcare facilities, were developed on a foundation of colonialism and racism constituted by the Indian Act (59). Furthermore, diversity exists within and between different Indigenous communities, suggesting barriers are likely unique to regional communities and experiences. A community-specific approach is likely required to enhance representation of visible minorities. Variations exist in beliefs and experiences among individual participants and between different communities or races; there is evidence that different barriers and strategies may be emphasized in one racial group over another (60). Knowing that there is no one-size-fits-all approach for all communities, it is critical to tailor strategies to the community of interest (40).

Future work should focus on identifying barriers and strategies in MSD research that reflects a broader range of visible minorities, for example Indigenous, South Asian, and East Asian groups living in Westernized countries. The intersectional experiences (i.e., the interconnectedness of social categorizations, such as race, gender, socioeconomic status, and other factors that are overlapping and interdependent) of minorities may not be reflected since only race was assessed. Future research should provide actionable steps for improving diversity in health research, with consideration of how intersections of race and other social determinants may influence participation.

## Conclusion

Compared to White counterparts, visible minorities experience a greater prevalence and disease severity of MSDs, including various forms of arthritis. Yet, visible minorities are largely underrepresented in clinical research. Underrepresentation in clinical research creates critical gaps in understanding underlying illness mechanisms, disease impacts, and treatment efficacy, which all may vary due to race-related factors. This scoping review sought to identify barriers to research participation by visible minorities; and highlight strategies implemented in MSD research to overcome these barriers. Among 28 studies included in the review, the most commonly cited barriers to research participation were mistrust, logistical barriers, and lack of awareness or understanding of research. Strategies for increasing diversity included ensuring benefit to participants, recruiting through sites serving the community of interest, and addressing logistical barriers. This scoping review provides health researchers with strategies for improving representation among participants and making MSD health research more equitable to better address the health of a diverse population.

## Data availability statement

The original contributions presented in the study are included in the article/Supplementary material, further inquiries can be directed to the corresponding author.

## Author contributions

DL, RA, DR, NI, SH, AB, and MM conducted the conception, design, analysis, and interpretation. DL, RA, DR, and MM conducted the article selection and data extraction. DL, RA, DR, NI, AB, and MM conducted the drafting and critical review for intellectual content. DL, RA, DR, NI, SH, AB, and MM conducted the approval of final manuscript. All authors contributed to the article and approved the submitted version.

## Funding

MM is supported by the Arthritis Society Stars Mid-Career Development Award funded by the Canadian Institute of Health Research-Institute of Musculoskeletal Health and Arthritis. This work was supported by Natural Sciences and Engineering Research Council of Canada (NSERC) Discovery grant (353715).

## Conflict of interest

The authors declare that the research was conducted in the absence of any commercial or financial relationships that could be construed as a potential conflict of interest.

## Publisher’s note

All claims expressed in this article are solely those of the authors and do not necessarily represent those of their affiliated organizations, or those of the publisher, the editors and the reviewers. Any product that may be evaluated in this article, or claim that may be made by its manufacturer, is not guaranteed or endorsed by the publisher.

## Footnotes

^1^Systemic Lupus Erythematosus is the most common form of lupus. Lupus is a chronic auto-immune disease.
