# Supplementary material for: Barriers and strategies for recruiting participants who identify as racial minorities in musculoskeletal health research: a scoping review
Source: Front Public Health. 2023 Aug 2;11:1211520. doi: 10.3389/fpubh.2023.1211520 (PMC10433765; doi:10.3389/fpubh.2023.1211520)
Supplement: Supplementary file 1 [file Table_1.docx]

Supplemental 1. Search strategies to identify papers on race, research participation and musculoskeletal disease.

| **Search Category** | **Key Terms** | ***EMBASE*** | ***MEDLINE*** | ***CINAHL*** | ***PsycInfo*** |
| --- | --- | --- | --- | --- | --- |
| Race | Race  racial*  ethnic*  minority  diversity  Underserved  Asian*  Black  African American  **African***  Chinese  Indigenous  Aboriginal/Aboriginee  Native American  Hispanic  **First Nation, Metis, Inuit**  **Latin***  **Arab***  **Filipino, Korean, Japanese**  **Indian/Pakistan*** | **Subject headings**  exp minority health/ or minority group/ or exp race difference/ or exp racism/ or exp ethnicity/ or exp "ethnic or racial aspects"/ or health disparity/ or health care disparity/ or ethnic group/ or exp population group/ or exp ancestry group/ or exp race/ or race difference/ or race relation/ or racism/  **Text words**  (Race or racial* or ethnic* or minorit* or divers* or underserved or Asian* or Black* or Chinese or Indigenous OR aborigin* or native adj American* or First adj Nation* or Inuit or Metis or Hispanic* or Latin* or African adj American* or African* or arab* or Filipino or Korean or Japanese).ti,ab,kw | **Subject headings**  exp minority health/ or minority group/ or exp racism/ or healthcare disparities/ or health status disparities/or exp population groups/ or exp continental population groups/ or exp ethnic groups/  **Text words**  (Race or racial* or ethnic* or minorit* or divers* or underserved or Asian* or Black* or Chinese or Indigenous OR aborigin* or native adj American* or First adj Nation* or Inuit or Metis or Hispanic* or Latin* or African adj American* or African* or arab* or Filipino or Korean or Japanese).ti,ab,kw | **Subject headings**  ((MH "Race Factors") OR (MH "Minority Groups") OR (MH "Racism") OR (MH "Race Relations") OR (MH "Indigenous Health"))  **Text words**  (Race or racial* or ethnic* or minorit* or divers* or underserved or Asian* or Black* or Chinese or Indigenous OR aborigin* or “native American*” or “First Nation*” or Inuit or Metis or Hispanic* or Latin* or “African American*” or African* or arab* or Filipino or Korean or Japanese) | **Subject Heading**  {Race and Ethnic Discrimination} OR {Minority Groups} OR {Minority Stress} OR {Marginalized Groups} OR {Race (Anthropological)} OR {Racial Identity} OR {Racial and Ethnic Differences} OR {Racial and Ethnic Groups} OR {Racial and Ethnic Attitudes} OR {Race and Ethnic Discrimination} OR {Racial Disparities} OR {Ethnic Diversity} OR {Ethnic Identity} OR {Ethnic Identity} OR {Multiracial} OR {Cultural Sensitivity} OR {Blacks} OR {African Cultural Groups} OR {Arabs} OR {Asians} OR {European Cultural Groups} OR {Indigenous Populations} OR {Latinos/Latinas} OR {Romanies} OR {Tribes} OR {Whites} OR {Culture (Anthropological)} OR {Cross Cultural Differences} OR {Cross Cultural Psychology} OR {Cross Cultural Treatment} OR {Antiracism} OR {Health Disparities} OR {Diversity} OR {Inclusion}  **Text words**  (Race or racial* or ethnic* or minorit* or divers* or underserved or Asian* or Black* or Chinese or Indigenous OR aborigin* or “native American*” or “First Nation*” or Inuit or Metis or Hispanic* or Latin* or “African American*” or African* or arab* or Filipino or Korean or Japanese) |
| Participation | Participat*  Recruit*  Enrollment/Enrolment  Underrepresent*  Under-represent*  Patient selection  Patient participation  Perception/Perceive  Engag*  Retention/retain*  Research subjects | **Subject headings**  Patient Participation/  Patient selection/  patient attitude/ or patient participation/ or refusal to participate/ or exp treatment refusal/  research subject/  **Text words**  (recruit* or participat* or engag* or enrol* or underrepresent* or under?represent* or perception* or perceive or retention or retain*).ti,kw. | **Subject headings**  "patient acceptance of health care"/ or patient participation/ or exp treatment refusal/ or exp Community Participation/ or Patient Selection/ or Research Subjects/  **Text words**  (recruit* or participat* or engag* or enrol* or underrepresent* or under?represent* or retention or retain* or perception*).ti,kw. | **Subject Heading**  (MH "Patient Selection") OR (MH "Research Subject Recruitment") OR (MH "Research Subjects+")  **Text words**  (recruit* or participat* or engag* or enrol* or underrepresent* or under#represent* or retention or retain* or * or perception* or perceive) | **Subject Heading**  {Treatment Barriers} OR {Client Participation} OR {Parental Involvement} OR {Client Participation} OR {Group Participation} OR {Membership} OR {Experimental Recruitment} OR {Experimental Subjects} OR {Client Attitudes}  **Text words**  Keywords or Abstract  recruit* or participat* or engag* or enrol* or underrepresent* or “under-represent*” or retention or retain* or perception* |
| Research | Research  Clinical trial  Health research  Trial  Study  Clinical  Epidemiology SH  Experiment | **Subject headings**  exp clinical trial/ or exp "clinical trial (topic)"/ or medical research/ or exp clinical research/ or rehabilitation research/ or exp human experiment/ or applied research/ or basic research/ or behavioral research/ or ethnographic research/ or evaluation research/ or interdisciplinary research/ or exp medical research/ or exp participatory research/ or population research/ or public health systems research/ or exp qualitative research/ or exp **epidemiology/ or** exp epidemiological data/  **Text words**  or (research or clinical or study or studies or trial* or experiment*)**.**ti,ab,kw. | **Subject headings**  exp clinical trials as topic/ or observational studies as topic/ or exp epidemiologic studies/ or pilot projects/ or exp epidemiological monitoring/ or evaluation studies as topic/ or Qualitative Research/ or exp behavioral research/ or biomedical research/ or health services research/ or exp human experimentation/ or rehabilitation research/ or exp community-based participatory research/ or exp empirical research/ or research design/  **Text words**  (research or clinical or study or studies or trial* or experiment*).ti,ab,kw. | **Subject headings**  (MH "Experimental Studies+") OR (MH "Clinical Trials+") OR (MH "Nonexperimental Studies+") OR (MH "Qualitative Studies+") OR (MH "Quantitative Studies") OR (MH "Retrospective Design") OR (MH "Population Health Management") **OR (MH "Epidemiological Research+")**  **Text words**  TI and AB:  research or clinical or study or studies or trial* or experiment* | **Subject Heading**  {Qualitative Methods} OR {Interdisciplinary Research} OR {Action Research} OR {Public Health Research} OR {Quantitative Methods} OR {Clinical Trials} OR {Randomized Controlled Trials} OR {Experimental Design} OR {Epidemiology} OR {Comorbidity} OR {Morbidity} OR {Research Quality}  **Text words**  Keywords and Abstract:  research or clinical or study or studies or trial* or experiment* |
| Type of health research (Musculoskeletal) | Musculoskeletal  Arthriti*  Rheumat*  Bone*  Joint disease  Lupus | **Subject headings**  exp musculoskeletal disease/ or exp musculoskeletal disease assessment/ or exp musculoskeletal function/ or exp musculoskeletal system/ or exp lupus erythematosus/ or exp scleroderma/ or rheumatology/ or exp arthritis/  or exp osteology/ or exp functional anatomy/ or physical medicine/ or  rehabilitation medicine/ or rehabilitation/ or athletic rehabilitation/ or functional training/ or muscle training/ or occupational therapy/ or rehabilitation care/  **Text words**  (arthriti* or musculoskeletal or joint adj disease or lupus or rheumat*).ti,ab,kw. | **Subject headings**  exp musculoskeletal system/ or exp Musculoskeletal Diseases/ or exp arthritis/ or osteology/ or occupational therapy/ or physical therapy specialty/ or rheumatology/ or exp "physical and rehabilitation medicine"/ or exp lupus erythematosus, cutaneous/ or exp lupus erythematosus, systemic/ or scleroderma, localized/ or exp scleroderma, systemic/ or exp Musculoskeletal Physiological Phenomena/  **Text words**  (arthriti* or musculoskeletal or joint adj disease or lupus or rheumat*).ti,ab,kw. | **Subject headings**  (MH "Musculoskeletal System+") OR (MH "Musculoskeletal System Physiology") OR (MH "Musculoskeletal Diseases+") OR (MH "Musculoskeletal Care (Saba CCC)+") OR (MH "Diagnosis, Musculoskeletal+") OR (MH "Musculoskeletal Alteration (Saba CCC)") OR (MH "Lupus Erythematosus, Cutaneous") OR (MH "Lupus Erythematosus, Systemic+") OR (MH "Rheumatology") OR (MH "Occupational Medicine") OR (MH "Osteopathic Medicine") OR (MH "Physical Medicine")  **Text words**  (arthriti* or musculoskeletal or “joint disease” or rheumat* or lupus) | **Subject headings**  {Musculoskeletal Disorders} OR {Bone Disorders} OR {Bruxism} OR {Joint Disorders} OR {Musculoskeletal System} OR {Arm (Anatomy)} OR {Bones} OR {Feet (Anatomy)} OR {Hand (Anatomy)} OR {Hips} OR {Jaw} OR {Joints (Anatomy)} OR {Leg (Anatomy)} OR {Muscles} OR {Spinal Column} OR {Tendons} OR {Arthritis} OR {Rheumatic Fever} OR {Osteopathic Medicine} OR {Lupus}  **Text words**  (arthriti* or musculoskeletal or “joint disease*” or “joint disorder*” or rheumat* or lupus) |
