# Supplementary material for: Barriers and strategies for recruiting participants who identify as racial minorities in musculoskeletal health research: a scoping review
Source: Front Public Health. 2023 Aug 2;11:1211520. doi: 10.3389/fpubh.2023.1211520 (PMC10433765; doi:10.3389/fpubh.2023.1211520)
Supplement: Supplementary file 2 [file Table_2.docx]

Supplemental 2. Resources for Increasing Diversity in Research (please note these do not form a comprehensive list).

- - - 1. Proposed Standards for Race-Based and Indigenous Identity Data Collection and Health Reporting in Canada(14)
      2. OHRC: Collecting human rights-based data (61)
      3. The Implicit Association Test: Identifying Personal Bias (62)
      4. PROGRESS-Plus Framework (41)
      5. Toolkit for increasing participation of Black Asian and Minority Ethnic Groups in Social and Healthcare research (63)
      6. Resources and Guides for Indigenous Research (64)
      7. The INCLUDE Ethnicity Framework for increasing inclusion in research (65)
      8. Meaningful Community Collaboration in Research (66)
      9. Principles of Community Engagement (39)
      10. An Antiracist Framework for Racial and Ethnic Health Disparities Research (67)
      11. Key Public Health Resources for Anti-Racism Action: A Curated List (68)
      12. Best Practices in Equity, Diversity and Inclusion in Research (69)
